# Author Correction: Multiscale structural control of thiostannate chalcogels with two-dimensional crystalline constituents

**DOI:** 10.1038/s41467-024-52647-1

**Published:** 2024-10-01

**Authors:** Thanh Duy Cam Ha, Heehyeon Lee, Yeo Kyung Kang, Kyunghan Ahn, Hyeong Min Jin, In Chung, Byungman Kang, Youngtak Oh, Myung-Gil Kim

**Affiliations:** 1 School of Advanced Materials Science & Engineering, Sungkyunkwan University, Suwon, 16491 Republic of Korea; 2 Department of Materials Science and Engineering, Korea University, Seoul, 02841 Republic of Korea; 3 Center for Sustainable Environment Research, Korea Institute of Science and Technology, Seoul, 02792 Republic of Korea; 4 Department of Organic Materials Engineering, Chungnam National University, Daejeon, 34134 Republic of Korea; 5 School of Chemical and Biological Engineering, and Institute of Chemical Processes, Seoul National University, Seoul, Republic of Korea; 6 Center for Correlated Electron Systems, Institute for Basic Science (IBS), Seoul, Republic of Korea; 7 Nuclear Chemistry Research Division, Korea Atomic Energy Research Institute, Daejeon, 34057 Republic of Korea

Correction to: *Nature Communications* 10.1038/s41467-022-35386-z, published online 23 December 2022

In this article, the funding from ‘the Technology Innovation Program (20004977) funded by the Ministry of Trade, Industry, & Energy (MOTIE, Korea).’ was omitted. The original article has been corrected.

