# Supplementary material for: Multiscale structural control of thiostannate chalcogels with two-dimensional crystalline constituents
Source: Nat Commun. 2022 Dec 23;13:7876. doi: 10.1038/s41467-022-35386-z (PMC9789151; doi:10.1038/s41467-022-35386-z)
Supplement: Supplementary file 1 — Supplementary Information [file 41467_2022_35386_MOESM1_ESM.docx]

**Supplementary Information**

**Multiscale structural control of tin sulfide-based chalcogel with two-dimensional crystalline constituents**

Thanh Duy Cam Ha^1,^[^‡^](https://pubs.rsc.org/en/content/articlelanding/2020/ta/c9ta11282k#fn2), Heehyeon Lee^2,3,^[^‡^](https://pubs.rsc.org/en/content/articlelanding/2020/ta/c9ta11282k#fn2), Kang Yeo Kyung^1^, Kyunghan Ahn^1^, Hyeong Min Jin^4^, In Chung^5,6^, Byungman Kang^7^, Youngtak Oh ^3*^ and Myung-Gil Kim ^1*^

^1^ School of Advanced Materials Science & Engineering, Sungkyunkwan University, Suwon 16491, Republic of Korea.

^2^ Department of Materials Science and Engineering, Korea University, Seoul 02841, Republic of Korea.

^3^ Center for Sustainable Environment Research, Korea Institute of Science and Technology, Seoul 02792, Republic of Korea.

^4^ Department of Organic Materials Engineering, Chungnam National University, Daejeon, 34134, Republic of Korea.

^5^ School of Chemical and Biological Engineering, and Institute of Chemical Processes, Seoul National University, Seoul, Republic of Korea.

^6^ Center for Correlated Electron Systems, Institute for Basic Science (IBS), Seoul, Republic of Korea.

^7^ Nuclear Chemistry Research Division, Korea Atomic Energy Research Institute, Daejeon 34057, Republic of Korea.

[^‡^](https://pubs.rsc.org/en/content/articlelanding/2020/ta/c9ta11282k#fn2) These authors contributed equally: Thanh Duy Cam Ha and Heehyeon Lee.

*Corresponding authors Email: [myunggil@skku.edu](mailto:myunggil@skku.edu); [ytoh@kist.re.kr](mailto:ytoh@kist.re.kr)

**Table of Contents:**

[**Supplementary Methods** 4](#_Toc117893584)

[**Supplementary Figures** 6](#_Toc117893585)

[Supplementary Figure 1. 6](#_Toc117893586)

[Supplementary Figure 2. 7](#_Toc117893587)

[Supplementary Figure 3. 8](#_Toc117893588)

[Supplementary Figure 4 9](#_Toc117893589)

[Supplementary Figure 5. 10](#_Toc117893590)

[Supplementary Figure 6 11](#_Toc117893591)

[Supplementary Figure 7 12](#_Toc117893592)

[Supplementary Figure 8 13](#_Toc117893593)

[Supplementary Figure 9 14](#_Toc117893594)

[Supplementary Figure 10 15](#_Toc117893595)

[Supplementary Figure 11 16](#_Toc117893596)

[Supplementary Figure 12 17](#_Toc117893597)

[Supplementary Figure 13 18](#_Toc117893598)

[Supplementary Figure 14 19](#_Toc117893599)

[Supplementary Figure 15 20](#_Toc117893600)

[Supplementary Figure 16 21](#_Toc117893601)

[Supplementary Figure 17 22](#_Toc117893602)

[Supplementary Figure 18. 23](#_Toc117893603)

[Supplementary Figure 19. 24](#_Toc117893604)

[Supplementary Figure 20 25](#_Toc117893605)

[Supplementary Figure 21. 26](#_Toc117893606)

[Supplementary Figure 22. 27](#_Toc117893607)

[Supplementary Figure 23. 28](#_Toc117893608)

[Supplementary Figure 24. 29](#_Toc117893609)

[Supplementary Figure 25 30](#_Toc117893610)

[Supplementary Figure 26 31](#_Toc117893611)

[Supplementary Figure 28. 33](#_Toc117893612)

[Supplementary Figure 29. 34](#_Toc117893613)

[Supplementary Figure 30. 35](#_Toc117893614)

[**Supplementary Tables** 36](#_Toc117893615)

[Supplementary Table 1 36](#_Toc117893616)

[Supplementary Table 2. 37](#_Toc117893617)

[Supplementary Table 3. 38](#_Toc117893618)

[Supplementary Table 4. 39](#_Toc117893619)

[Supplementary Table 5. 40](#_Toc117893620)

[**Supplementary References** 41](#_Toc117893621)

Supplementary Methods

**g-Factor calculation of NMSC via electron paramagnetic resonance (EPR) analysis**

The g-factor can be calculated from *ν* (GHz) and *B_o_* (gauss, G) using Eqn. 1, where *h*, *v*, *β*, and *B_o_* correspond to the Planck constant (6.626 × 10^-34^ J s), frequency (9.64 GHz = 9640 MHz in the *X*-band klystron range of 8.8−9.6 GHz), Bohn magnetron constant (9.274E−28 J G^-1^) and magnetic field moment for each NMSC (NMSC-1: 3418 *G*, NMSC-2: 3423.5 *G*, and NMSC-3: 3430.8 *G*), respectively.

$g=\frac{hv}{\beta B_{0}}=0.7145\frac{v\left( MHz \right)}{B_{0}\left( Gauss \right)}$ (1)

**Water contact angle measurement**

The wettability of the surface of NMSC-1 is investigated by water contact angle measurement. The 1 g of dried NMSC-1 was grinded and pelletized under the pressure of 350 bar to make the flatten surface of sample. The water drop is dropped on the surface of NMSC-1 pellet and the angle of water drop on the surface is measured.

**Competitive ion experiment**

The Na^+^-Cs^+^ and Na^+^-Sr^2+^ dual ion solutions are prepared at two different concentration ratios. The Cs^+^ initial concentration of solutions is 10 ppm. The Na^+^ initial concentration is variable at 10 ppm and 100 ppm in Na^+^-Cs^+^ dual ion solution batches (respectively, Na^+^:Cs^+^= 1:1 and Na^+^:Cs^+^= 10:1). The Sr^2+^ initial concentration of solutions is 10 ppm. The Na^+^ initial concentration is variable at 10 ppm and 100 ppm in Na^+^-Sr^2+^ dual ion solution batches (respectively, Na^+^:Sr^2+^= 1:1 and Na^+^:Sr^2+^= 10:1).

**Isoelectric point (Point of zero charge, PZC) measurement by the Salt Addition Method**

Isoelectric point values for NMSC samples are determined in 0.1 M NaNO_3_ solution at 298 K. In this method, the sample (0.01 g) and 0.1 M NaNO_3_ (40 mL) are mixed in various conical tubes using ultrasonic bath in 10 min. The pH of the suspension is then adjusted to an initial pH value of 2, 3, 4, 5, 6, 7, 8, 9, 10, and 11 using either 0.1 M HNO_3_ or 0.1 M NaOH solutions and an OHAUS pH-meter model ST300. Each tube then is vigorously agitated in a shaker bath for 24 h. Then, the final pH of each suspension is measured carefully. The ΔpH (the difference between final and initial pH) values are plotted against the initial pH values. The initial pH at which ΔpH is zero was taken to be the PZC.

Supplementary Figures


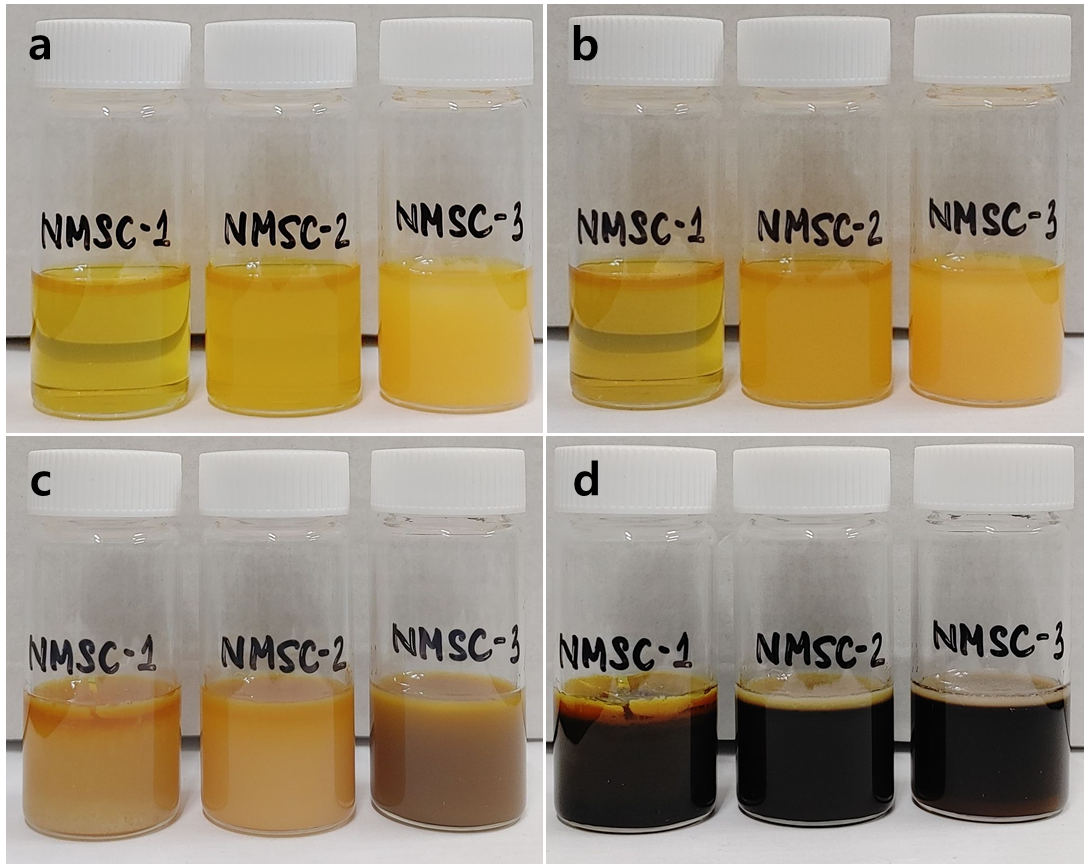


Supplementary Figure 1. Gelation process of NMSC samples. **a** As-prepared, **b** 15 min, **c** 12 h, and **d** 7 days.

**
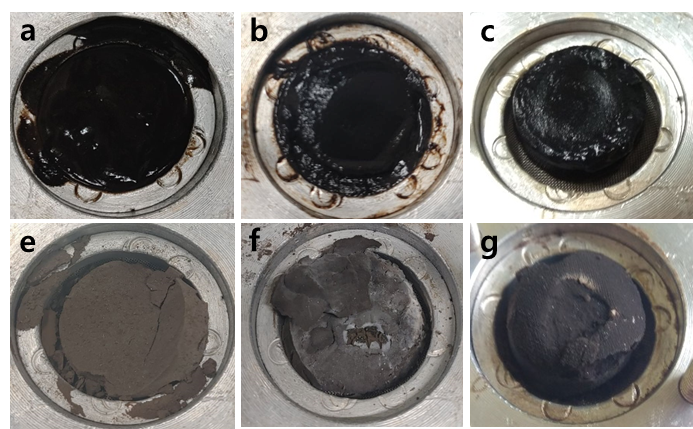
**

Supplementary Figure 2. Optical images of **a, e** NMSC-1, **b, f** NMSC-2, and **c, g** NMSC-3 before and after critical point drying (CPD).

In the optical images of NMSC samples (Supplementary Fig. 2), the rigidity of wet gel was directly proportional to the added amount of manganese. Overall, the physical shape of wet gel was maintained after critical point drying process.


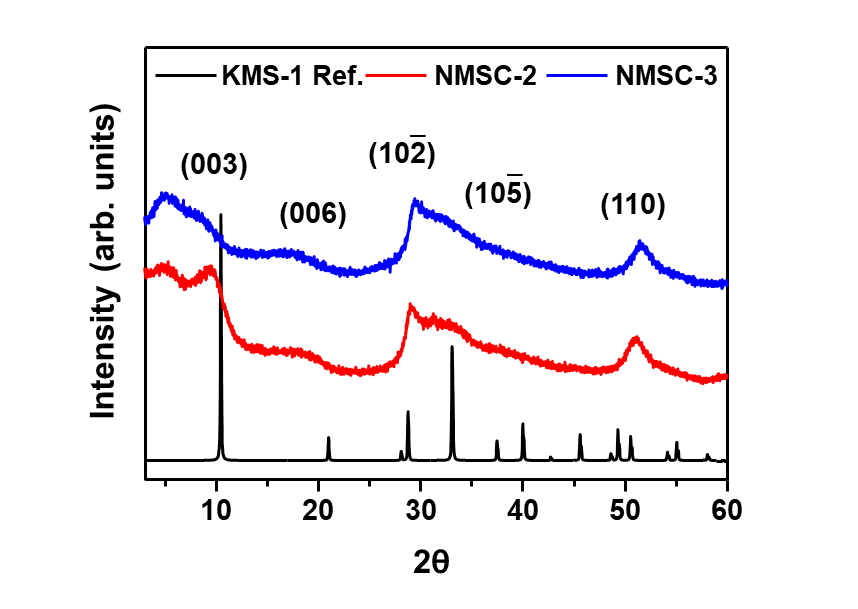


Supplementary Figure 3. **Crystal-structure similarity between KMS-1 and NMSCs.** Source data are provided as a Source Data file.

Comparison of the X-ray diffraction patterns of NMSC-2 and NMSC-3 to those of the KMS-1 (K_1.90_Mn_0.95_Sn_2.05_S_6_, ICSD #260314) bulk material. NMSC peak intensity is inversely proportional to Mn content^1^.


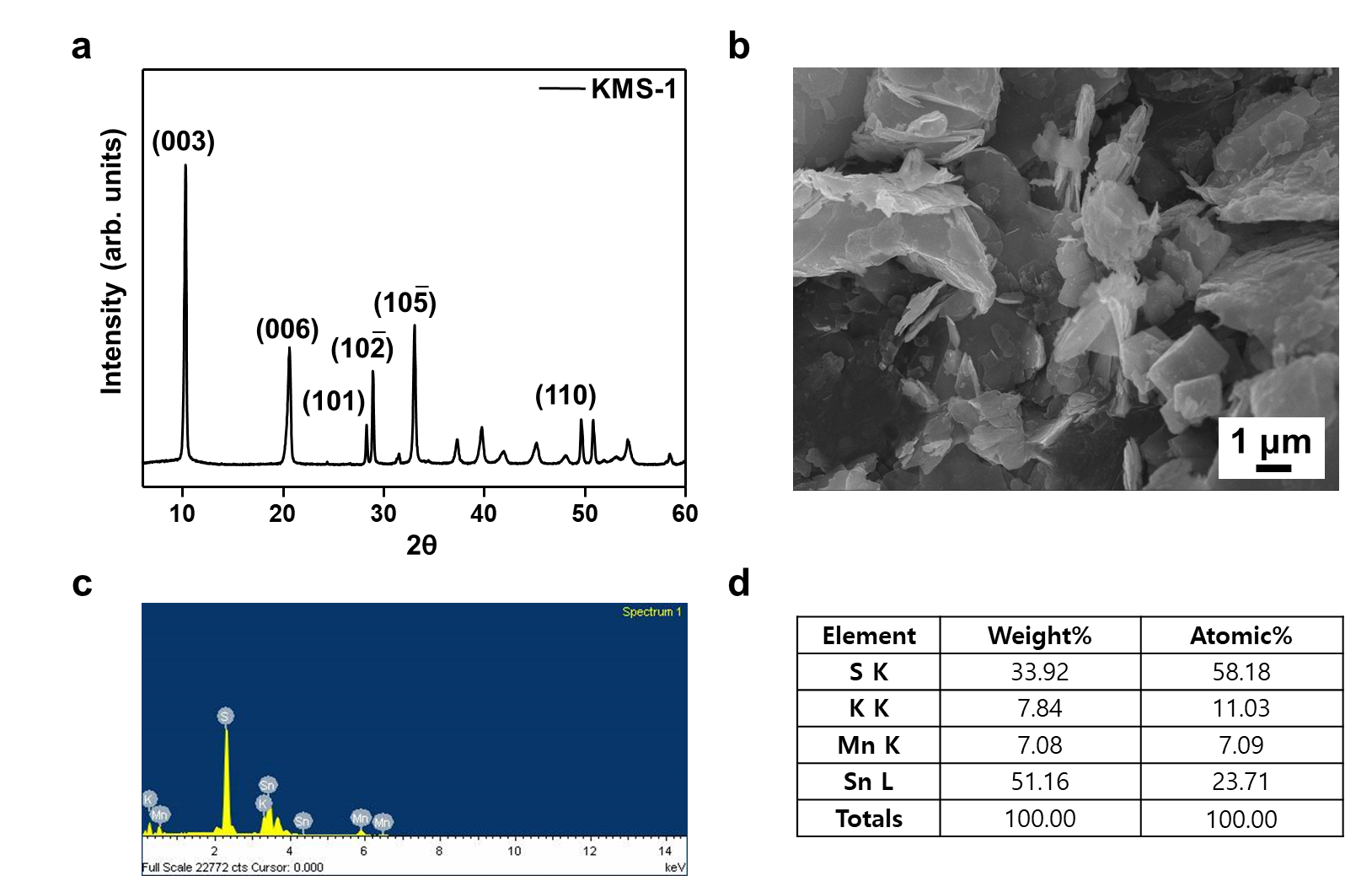


Supplementary Figure 4**. Characterizations of KMS-1. a** X-ray diffraction and **b** FE-SEM of bulk KMS-1 material reveal a 2D crystal structure with specific planes. **c, d** Elemental ratio of KMS-1 measured by EDS for K, Mn, Sn, and S, confirming the stoichiometry of layered metal sulfide K_1.90_Mn_0.95_Sn_2.05_S_6_ (ICSD #260314)^2^. Source data are provided as a Source Data file.


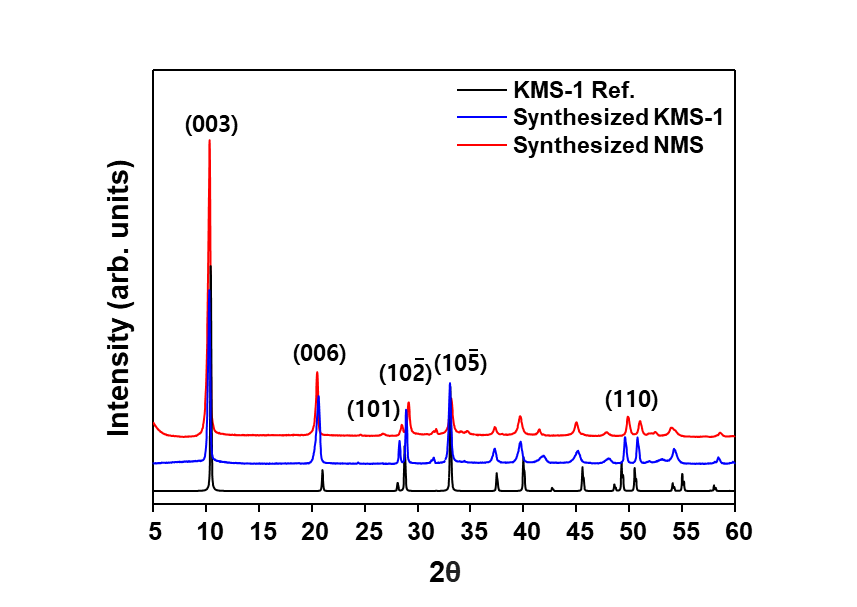


Supplementary Figure 5. X-ray diffraction of sodium manganese tin sulfide (NMS) powder material. Source data are provided as a Source Data file.
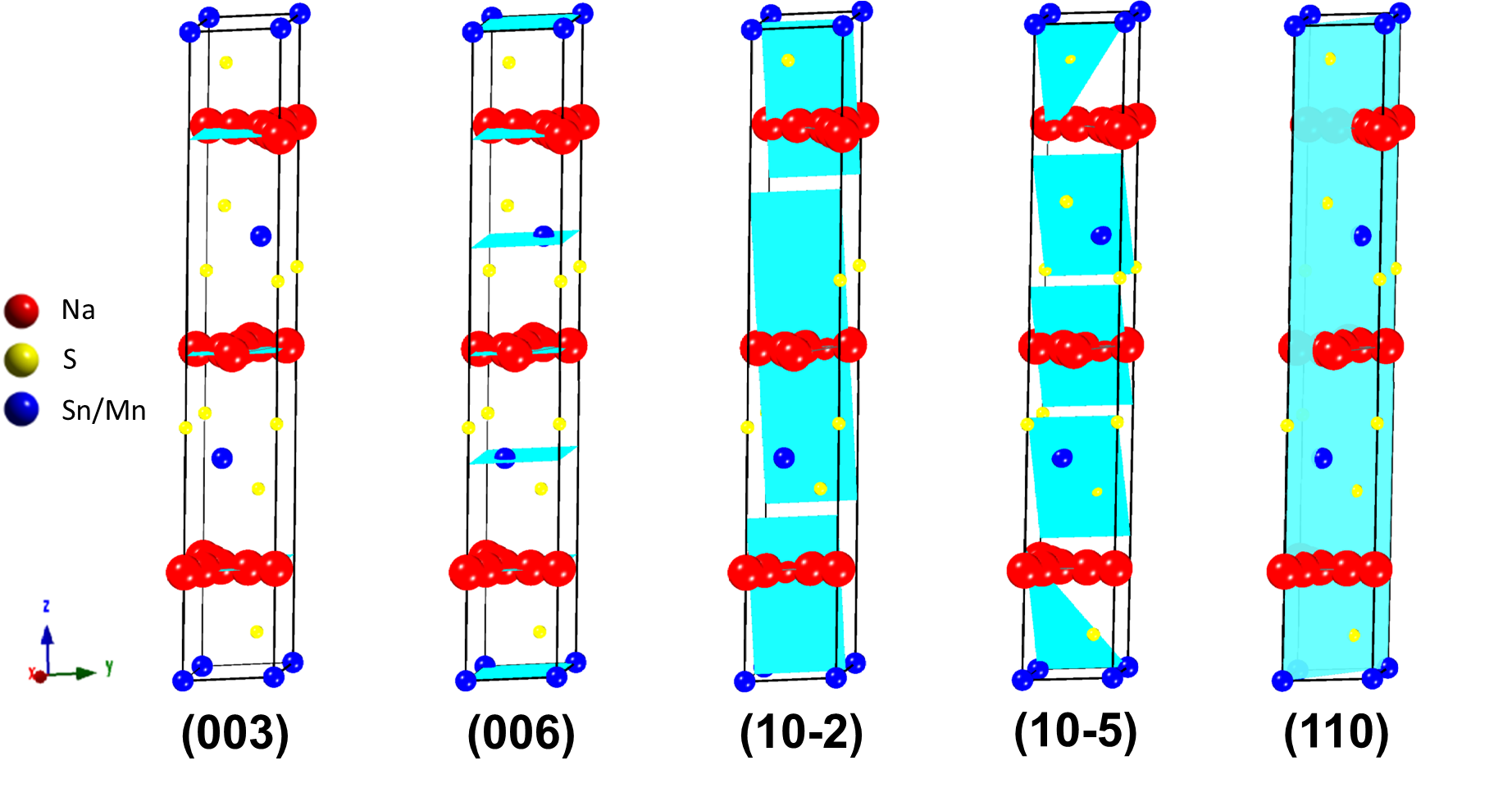


Supplementary Figure 6**.** ***R***$\bar{\boldsymbol{3}}$***m* crystal structure.** The specific representative planes of NMSCs according to the peak positions in X-ray diffraction (XRD) of sodium manganese tin sulfide powder (NMS) and KMS-1.


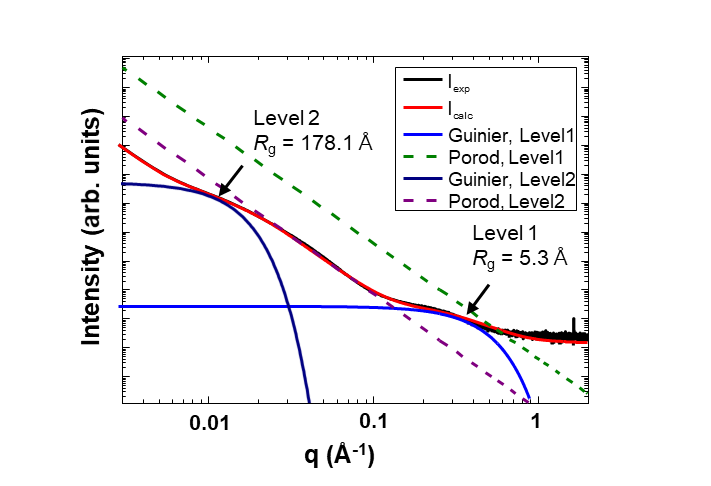


Supplementary Figure 7**.** **Small scattering angle analysis.** Small angle X-ray scattering (SAXS) profile of NMCS-1. Source data are provided as a Source Data file.


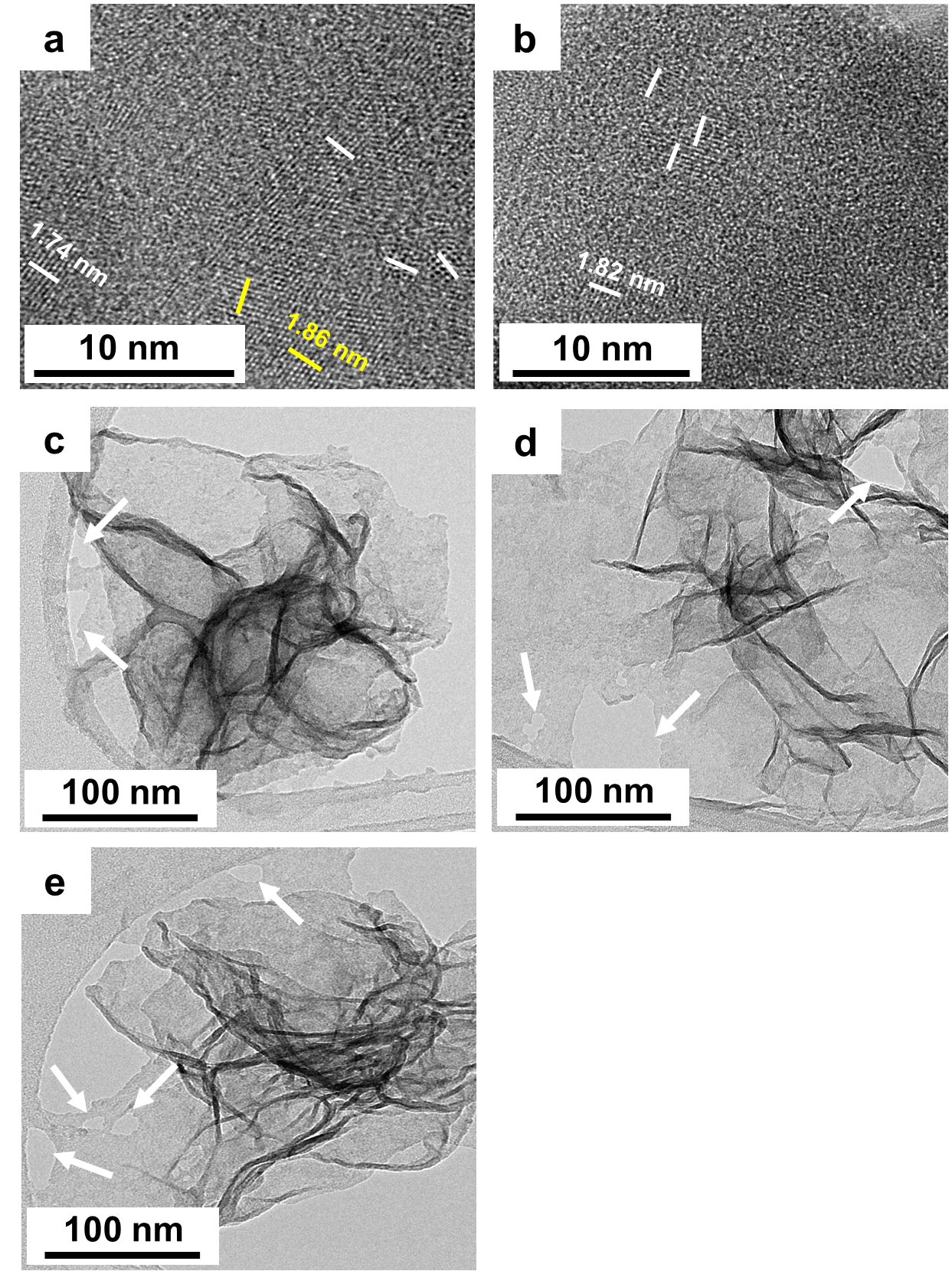


Supplementary Figure 8**.** **Pore-structure analysis of stacked NMSC layers.** The multilayered and intraparticle meso-porosity of NMSCs in the range 30–50 nm (white arrow) **a**, **c** NMSC-1, **b**, **d** NMSC-2, and **e** NMSC-3^3^.


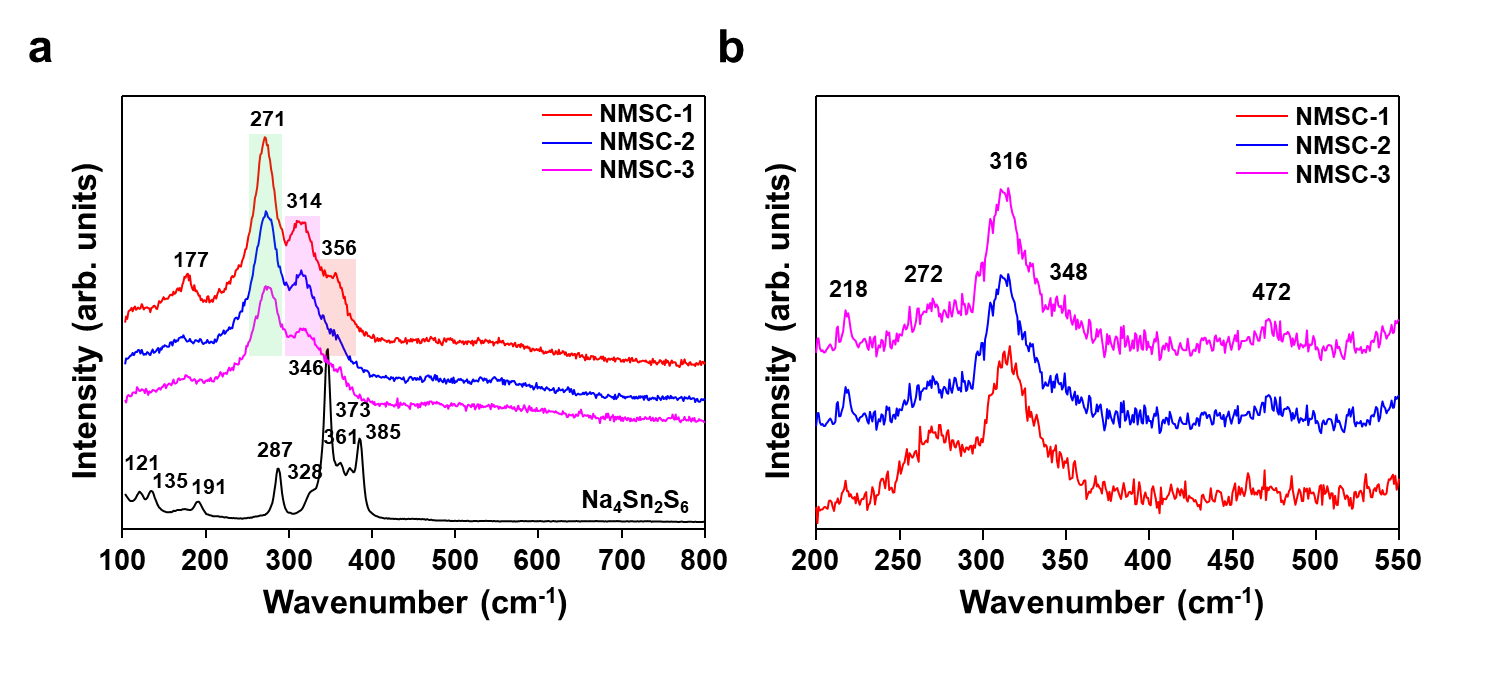


Supplementary Figure 9**.** **Chemical bonding-nature analysis of NMSC.** Raman spectroscopy analysis of Na_4_Sn_2_S_6_ and NMSC at different [Sn_2_S_6_]^4-^:Mn^2+^ ratios at wavelength **a** 785 and **b** 532 nm^4, 5^. Source data are provided as a Source Data file.


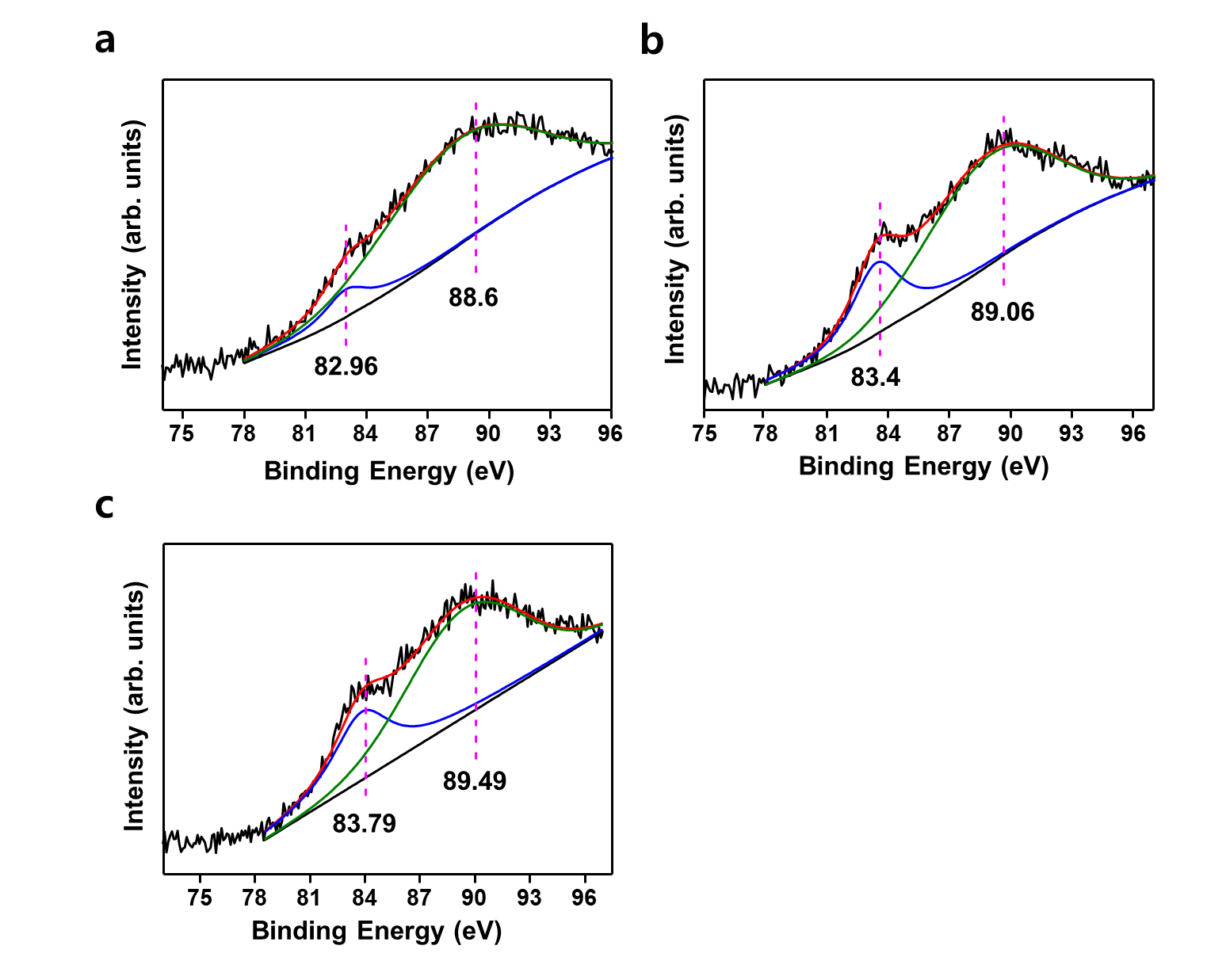


Supplementary Figure 10**.** **XPS curves of manganese in NMSCs.** XPS of **a** NMSC-1, **b** NMSC-2, and **c** NMSC-3 for Mn 3*s*. Source data are provided as a Source Data file.

**
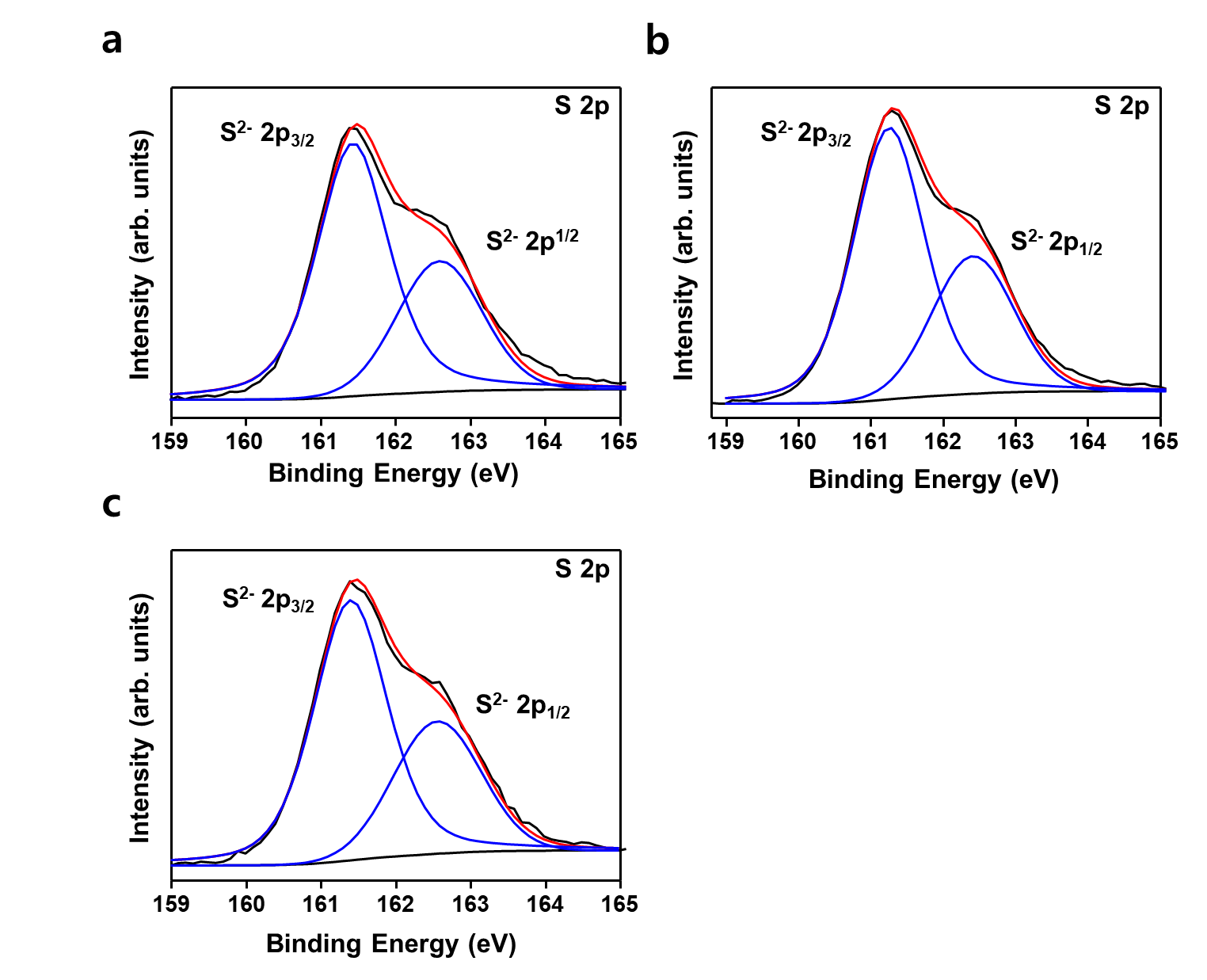
**

Supplementary Figure 11**.** **XPS curves of sulfur in NMSCs.** XPS of **a** NMSC-1, **b** NMSC-2, and **c** NMSC-3 for S 2*p*. Source data are provided as a Source Data file.


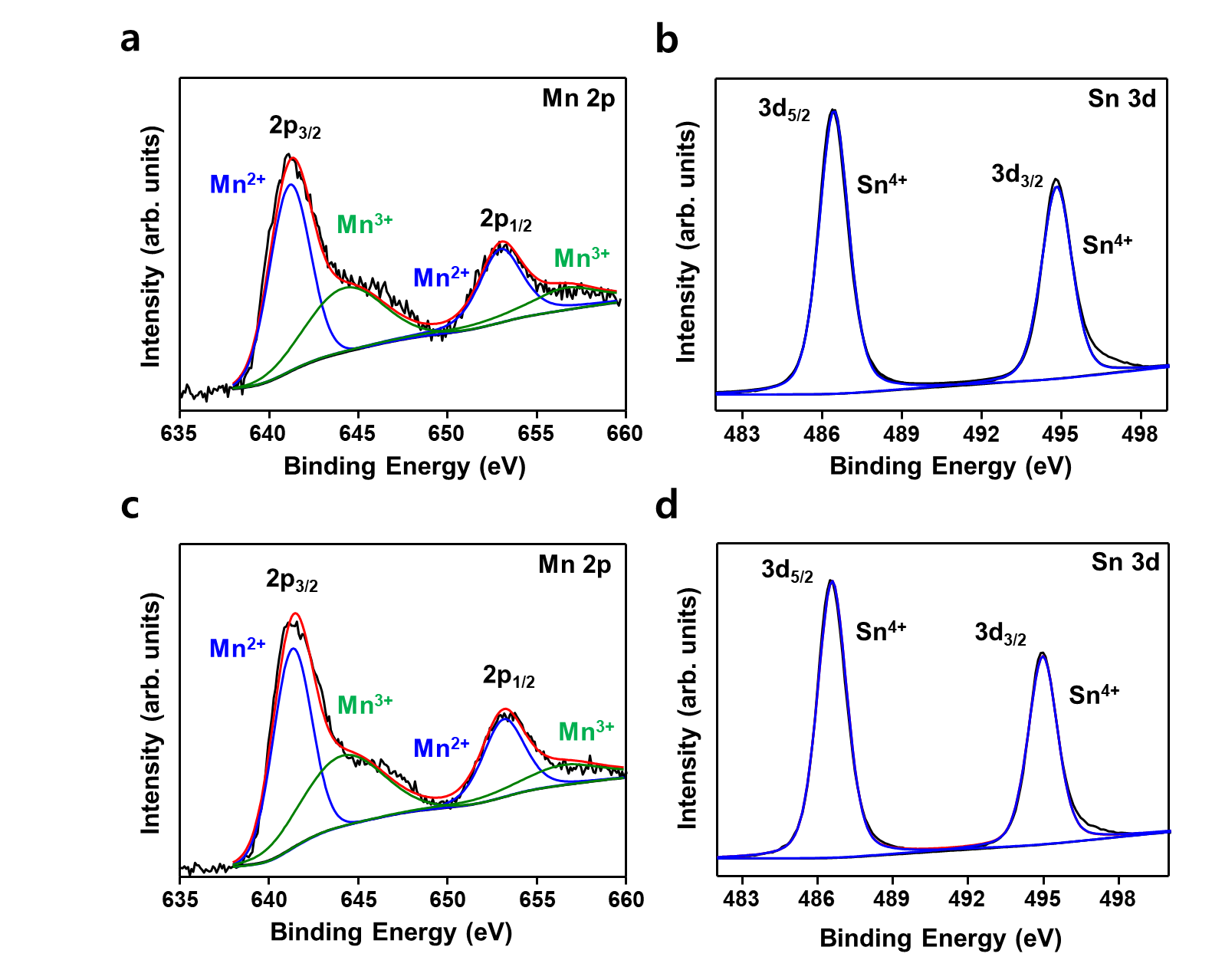


Supplementary Figure 12**.** **XPS curves of Mn and Sn in NMSCs.** XPS analysis of **a**, **b** NMSC-2 and **c**, **d** NMSC-3 for Mn 2*p* and Sn 3*d*. Source data are provided as a Source Data file.

The XPS spectra in Supplementary Fig. 12 indicate no variation in the oxidation state of Mn and Sn with the [Sn_2_S_6_]^4-^:Mn^2+^ ratios. However, it shows a decrease in the contribution of Mn^2+^ in oxidation state of the Mn^2+^ and Mn^3+^ mixture, which are 1.5:1 (NMSC-2) and 1.3:1 (NMSC-3) area ratios, respectively.


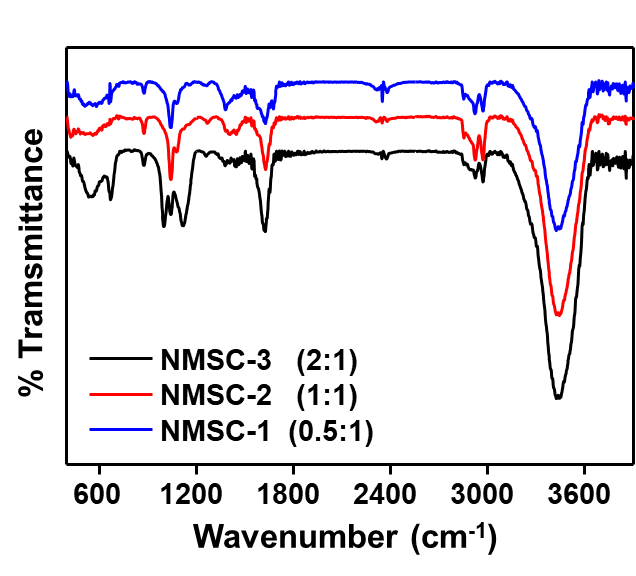


Supplementary Figure 13**. Functional group analysis of NMSCs.** Source data are provided as a Source Data file.

FT-IR of NMSC shows the presence of the acetate ion in the range 1000–3750 cm^-1^; the peak intensity is proportional to the [Sn_2_S_6_]^4-^:Mn^2+^ ratio with increasing amounts of the Mn(OOCCH_3_)_2_ precursor^6^.


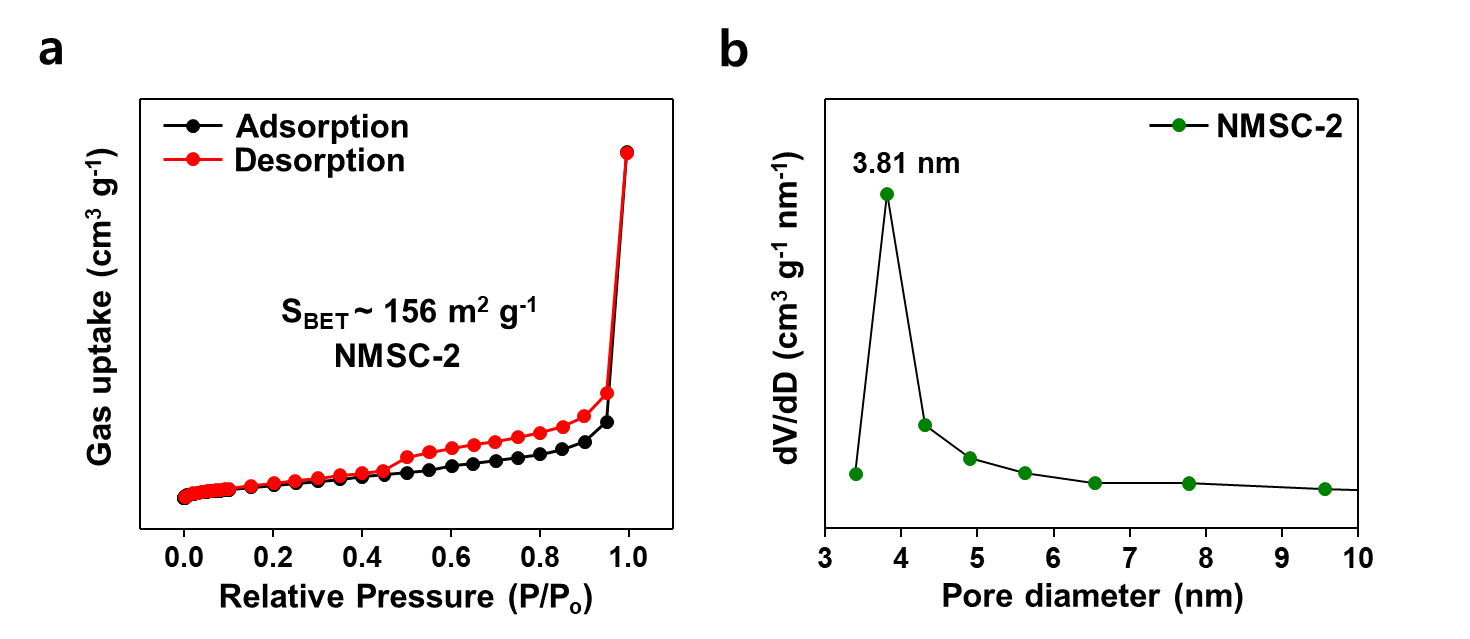


Supplementary Figure 14**. Pore-structure analysis of NMSC-2.** Brunauer–Emmett–Teller (BET) measures for **a** specific surface area and **b** pore size distribution of NMSC-2. Source data are provided as a Source Data file.


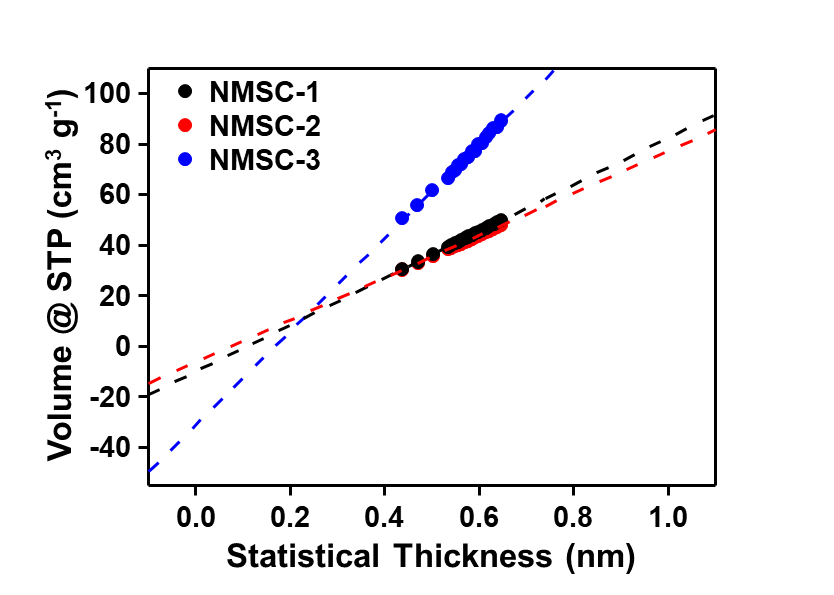


Supplementary Figure 15**. Micropore formation in NMSCs.** T-plot method for determining the presence of micropores in NMSCs. Source data are provided as a Source Data file.


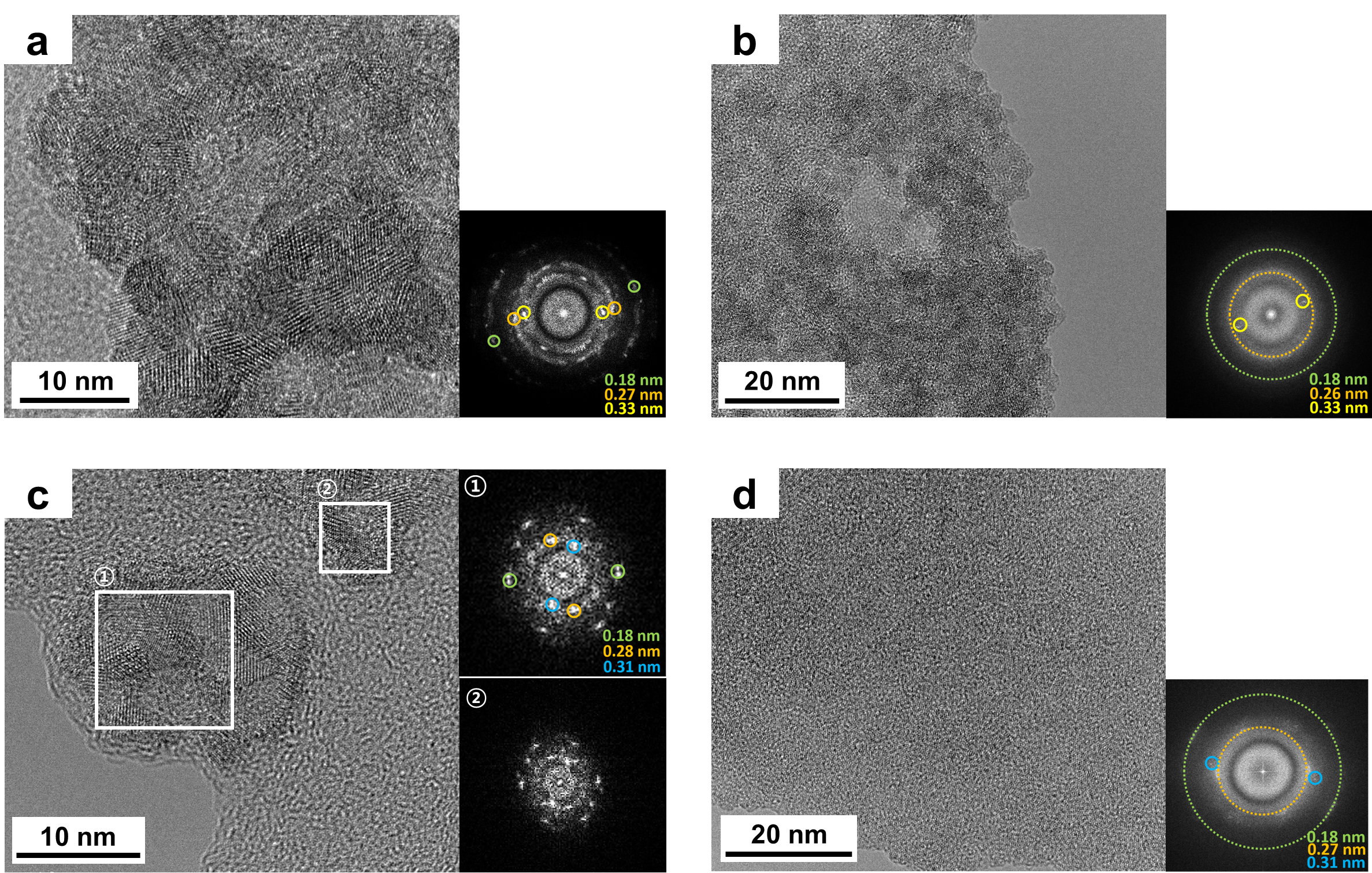


Supplementary Figure 16**. Layer formation in NMSC-1 during gelation.** HRTEM images and FFT mode analyses during the gelation of NMSC-1. **a** Na_4_Sn_2_S_6_ solution in formamide (FA). **b-d** The mixed Na_4_Sn_2_S_6_ and Mn(CH_3_COO)_2_ solution in FA after **b** 0, **c** 2.5, and **d** 60 h.


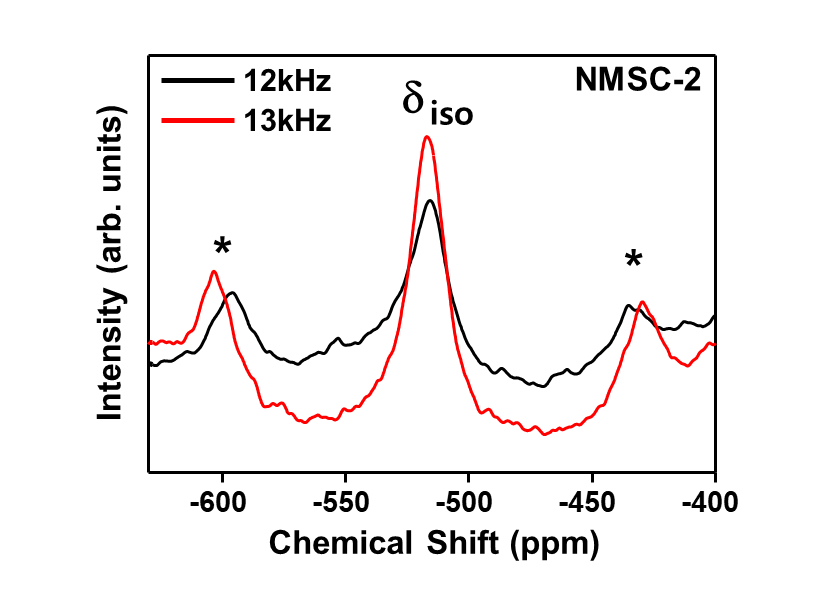


Supplementary Figure 17**. Octahedral coordination transformation of NMSC via ^119^Sn solid-state NMR analysis.** Source data are provided as a Source Data file.

^119^Sn MAS NMR spectra of NMSC-2 with different spinning rates: 12 and 13 kHz, which display a single peak at 520 ppm with no change in the chemical shift compared to other peaks, with various spinning rates. The peak at 520 ppm corresponds to the isotropic chemical shift of the octahedral Sn^4+^ cluster^7^.


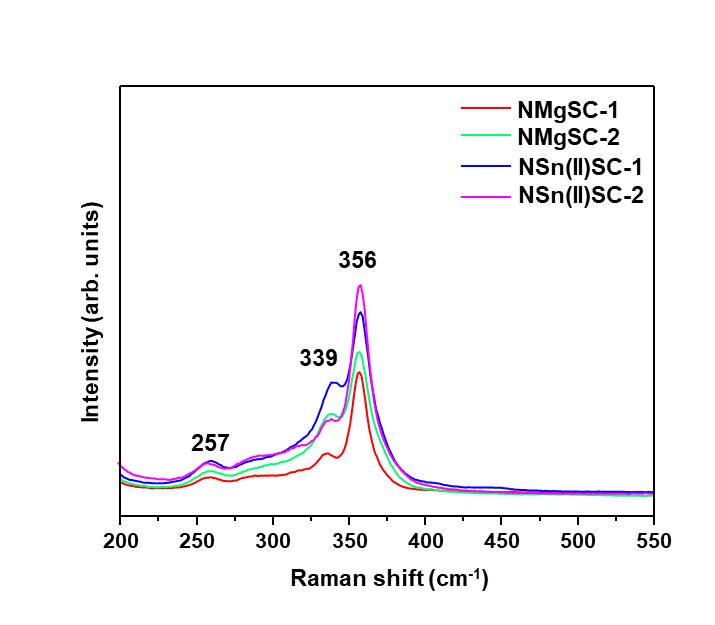


Supplementary Figure 18. **Chemical bonding-nature of NMgSC and NSn(II)SC sample**s. Raman spectroscopy analysis of NMgSC and NSn(II)SC aerogels at different [Sn_2_S_6_]^4-^:Mg^2+^/Sn^2+^ ratios. Peaks at 257, 339 and 356 cm^-1^ correspond to Sn_2_S_2_ ring and Sn-S octahedral vibrations at 532 nm. Source data are provided as a Source Data file.


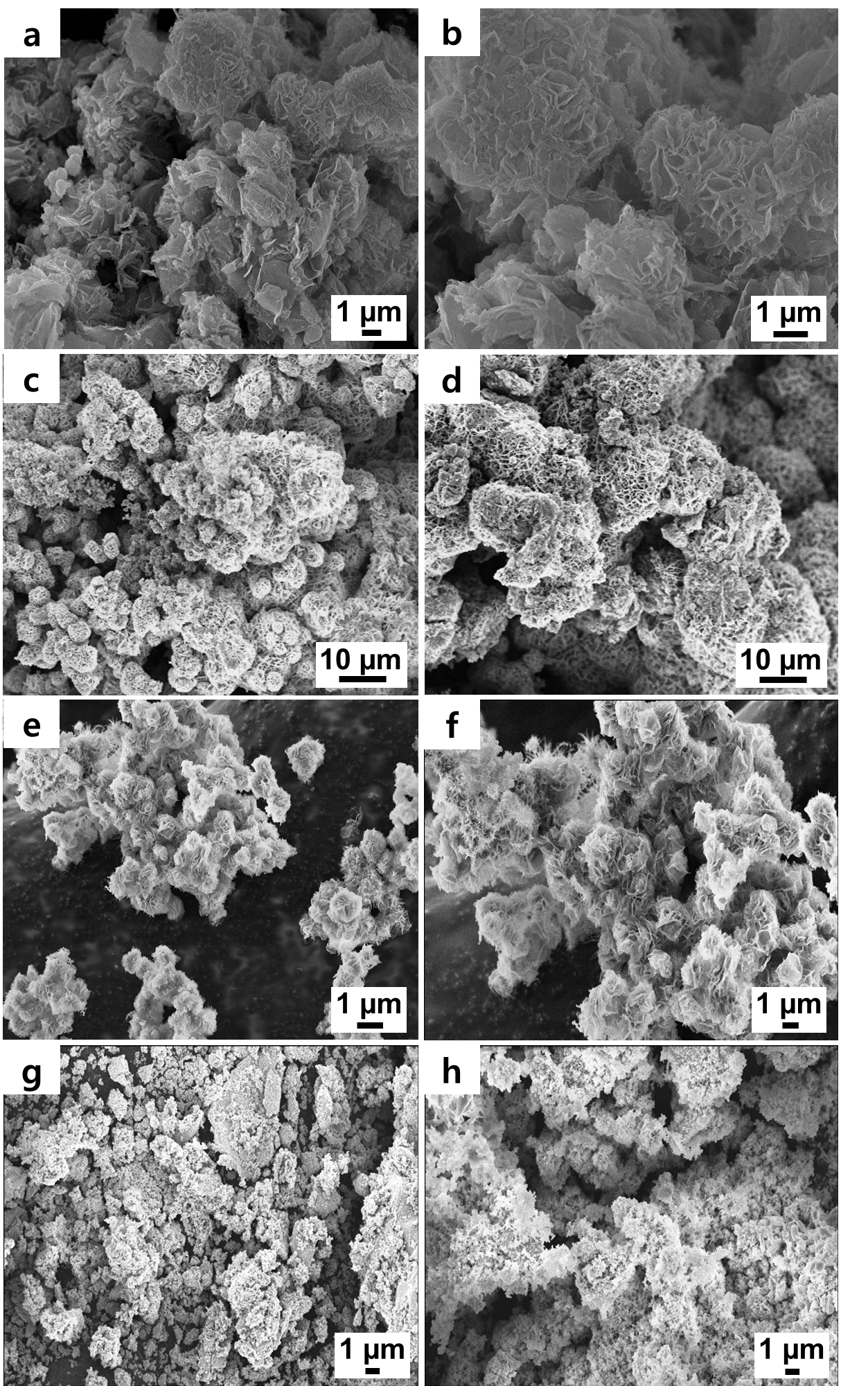


Supplementary Figure 19. **Morphological analysis of NMgSC and NSn(II)SC aerogel.** FE-SEM results of **a-b** NMgSC-1 (Mg^2+^:Sn_2_S_6_ = 0.5:1), **c-d** NMgSC-2 (Mg^2+^:Sn_2_S_6_ = 2:1), **e-f** NSn(II)SC-1 (Sn^2+^:Sn_2_S_6_ = 0.25:1) and **g-h** NSn(II)SC-2 (Sn^2+^:Sn_2_S_6_ = 2:1).


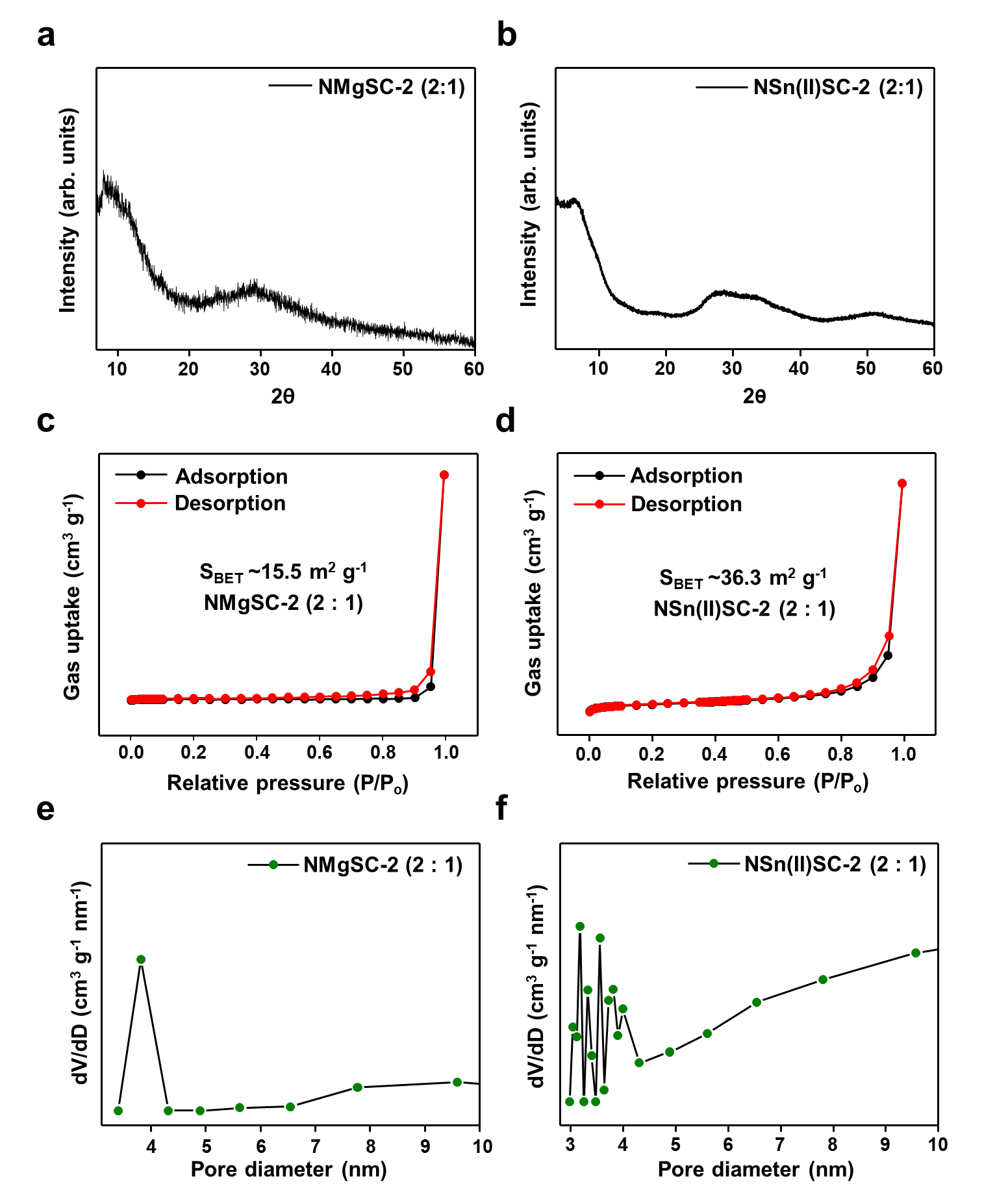


Supplementary Figure 20**.** **Crystal structure and pore characteristics of thiostannate chalcogels with high metal ion-linker content.** The crystal structure (PXRD) and porosity characteristics (BET and BJH) of various layered chalcogenide Na-Mg-Sn-S and Na-Sn(II)-Sn(IV)-S aerogels with high linker content. **a**, **c**, and **e** Na-Mg-Sn-S (Mg^2+^:Sn_2_S_6_ = 2:1, NMgSC-2) and **b**, **d**, and **f** Na-Sn(II)-Sn(IV)-S (Sn^2+^:Sn_2_S_6_ = 2:1, NSn(II)SC-2). Source data are provided as a Source Data file.


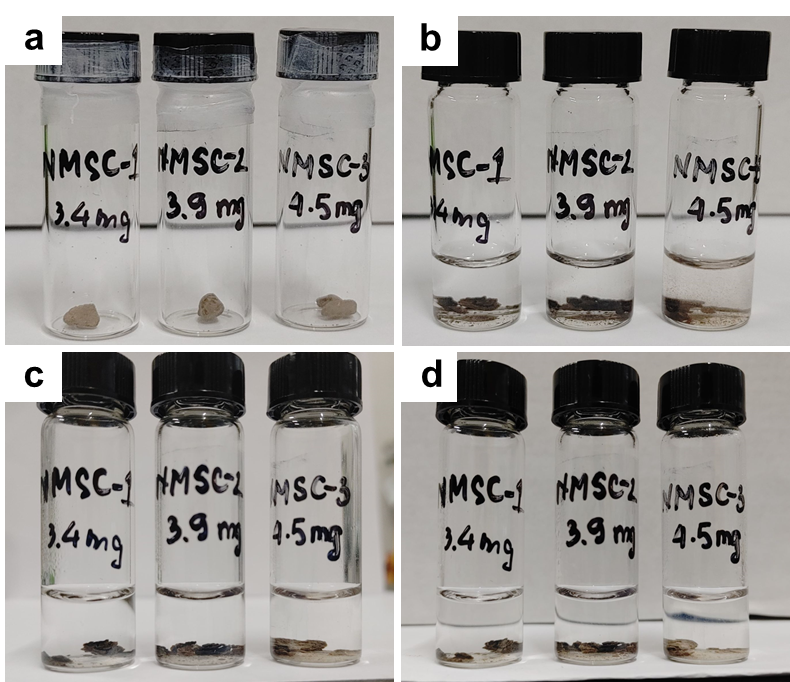


Supplementary Figure 21. **Stability of chalcogels during adsorption process in consideration time.** **a** Dried gel chunks. **b** After adding 2 mL Cs^+^ solution (100 ppm), **c** 12 h, and **d** 7 days.


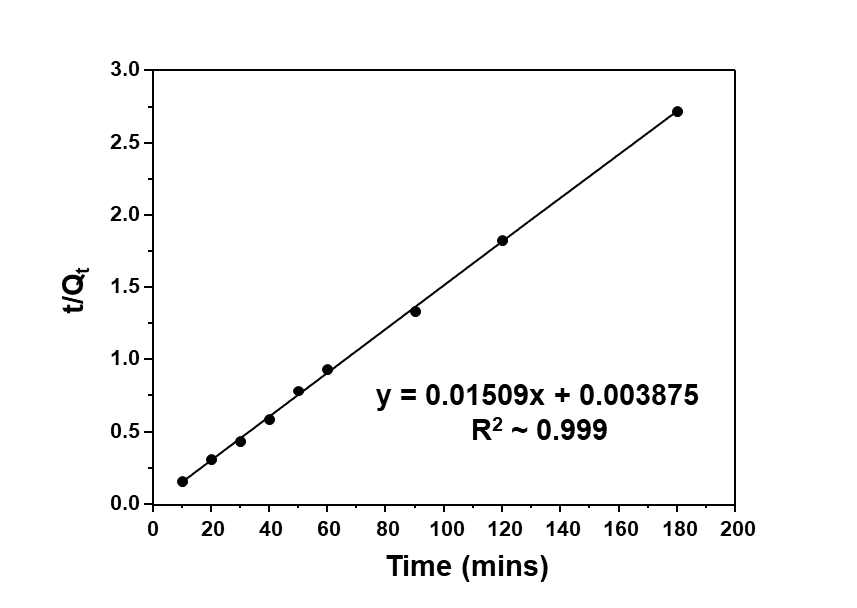


Supplementary Figure 22. **Kinetic analysis of NMSC-1 for Sr^2+^ ion exchange.** Raw adsorption kinetic (Q*t* vs. time) of NMSC-1 for Sr^2+^-exchanged (black line) described by pseudo-second-order fitting over the full equilibrium period (3 h). Source data are provided as a Source Data file.


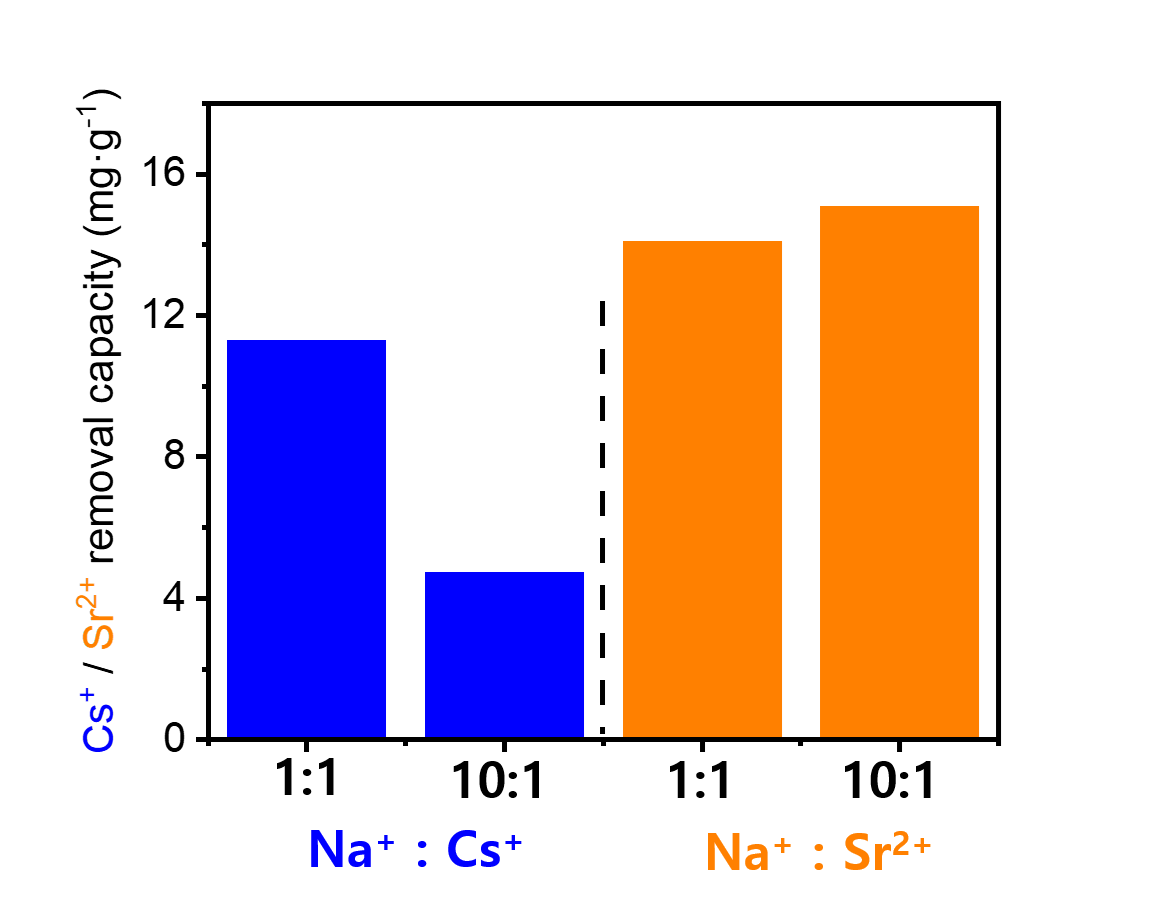


Supplementary Figure 23. Selective removal capacity of NMSC-1 in the Na^+^-Cs^+^ and Na^+^-Sr^2+^ co-existing solution at the different ratio. (Cs^+^ initial concentration: 10 ppm, Sr^2+^ initial concentration: 10 ppm). Source data are provided as a Source Data file.

**
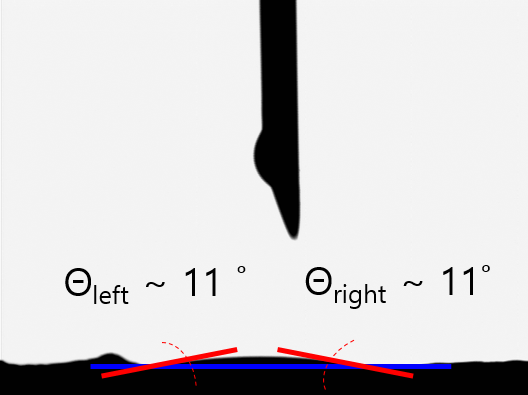
**

Supplementary Figure 24. Water Contact-angle measurement of NMSC-1.


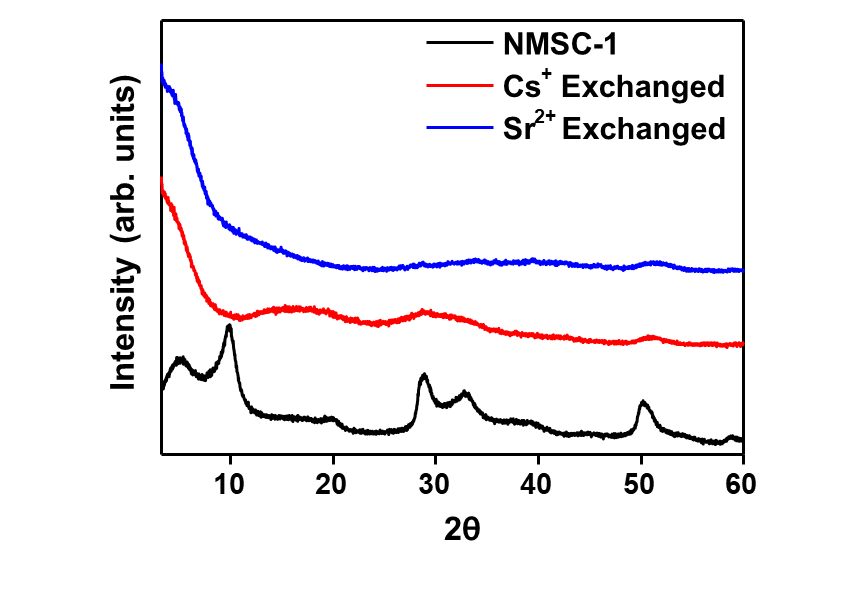


Supplementary Figure 25**.** **Crystal structure of NMSC-1 before and after radionuclide adsorption.** X-ray diffraction patterns of pristine, Cs^+^- and Sr^2+^-exchanged NMSC-1 samples. Source data are provided as a Source Data file.


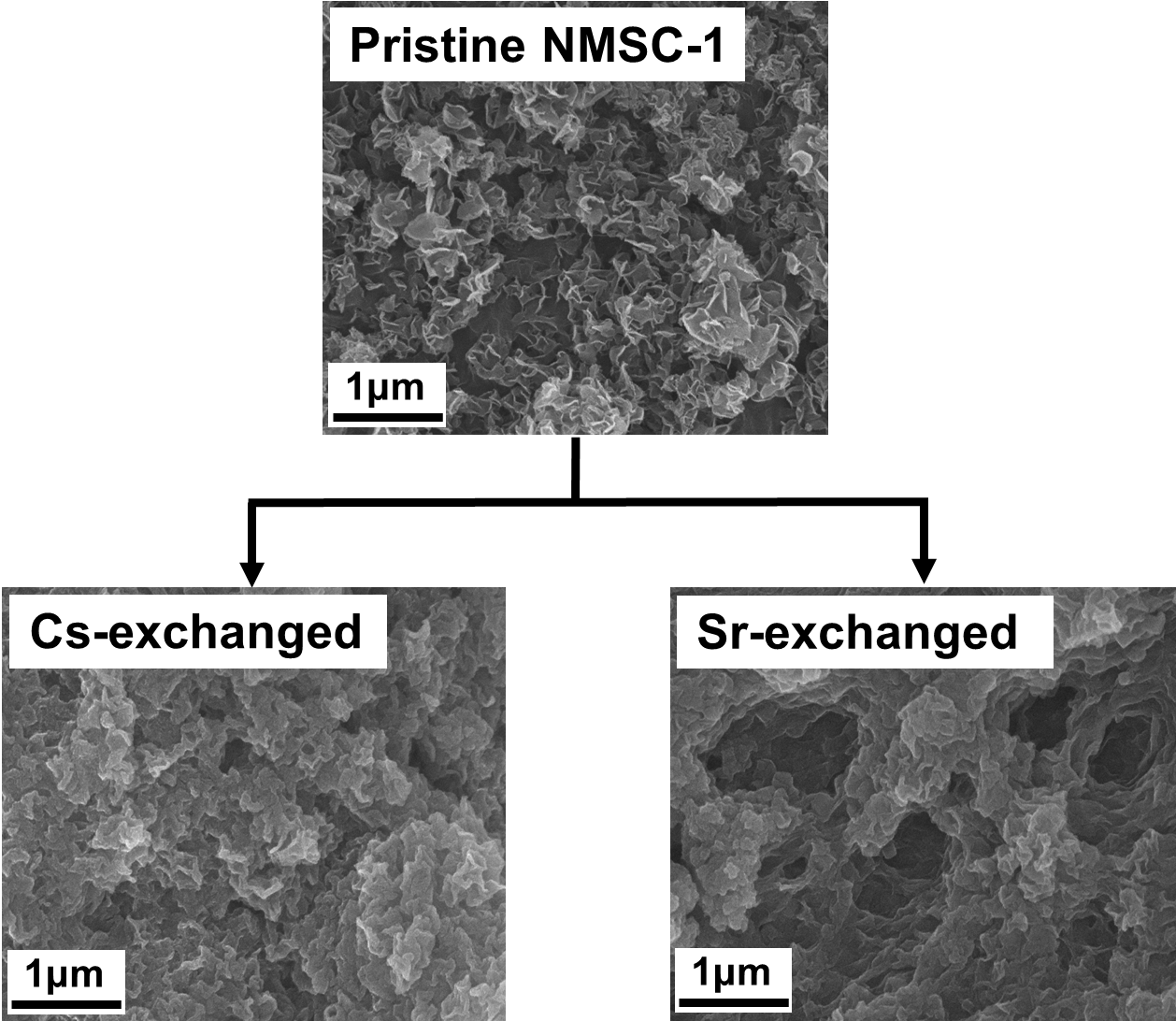


Supplementary Figure 26**. Morphological analysis of NMSC-1 before and after radionuclide adsorption.** FE-SEM images of pristine, Cs^+^- and Sr^2+^-exchanged NMSC-1 samples.


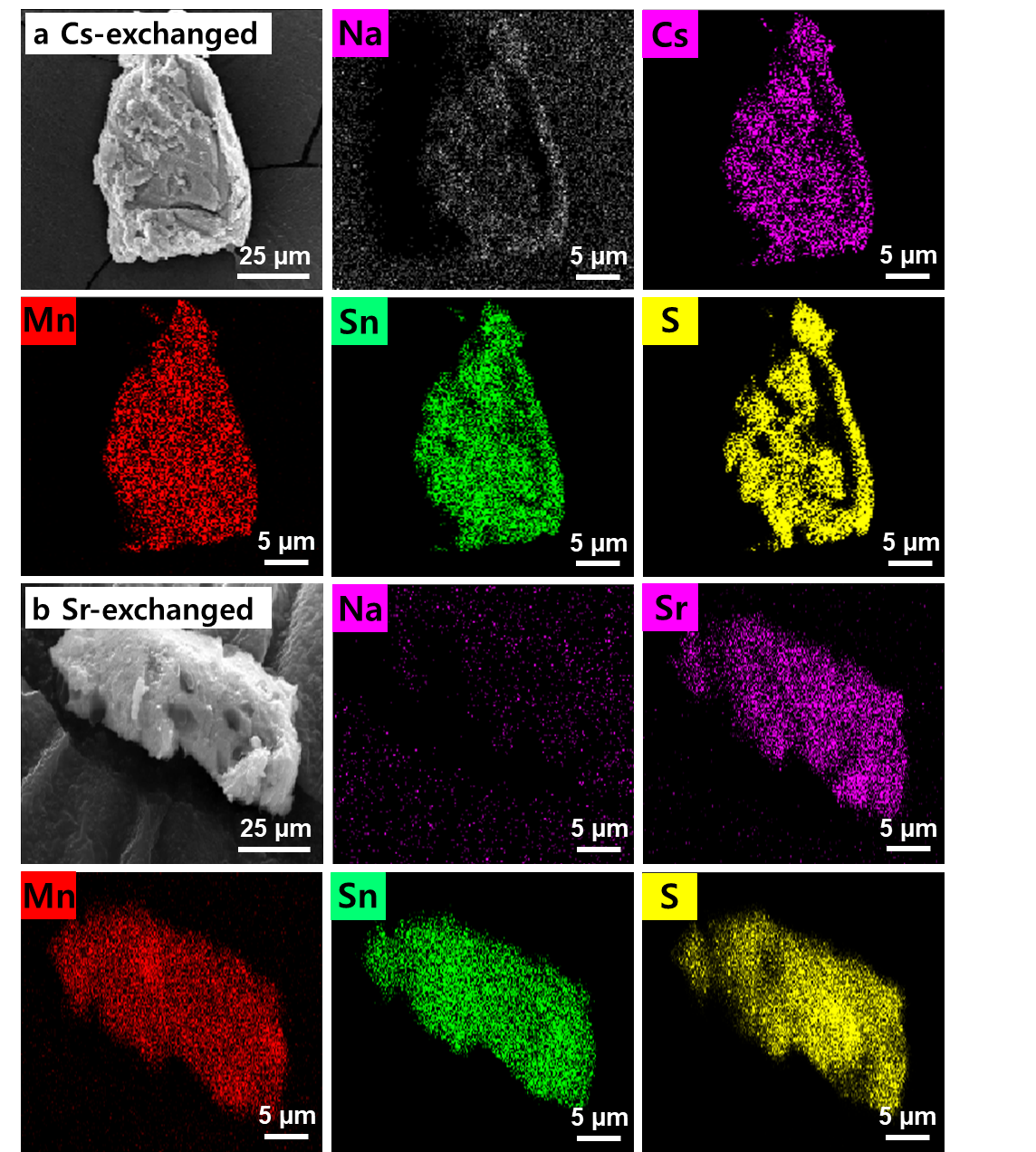


**Supplementary Figure 27. EDS mapping of NMSC-1 after ion exchanges.** **a** Cs^+^ and **b** Sr^2+^ include Na, Cs, Sr, Mn, Sn, and S elements.

**
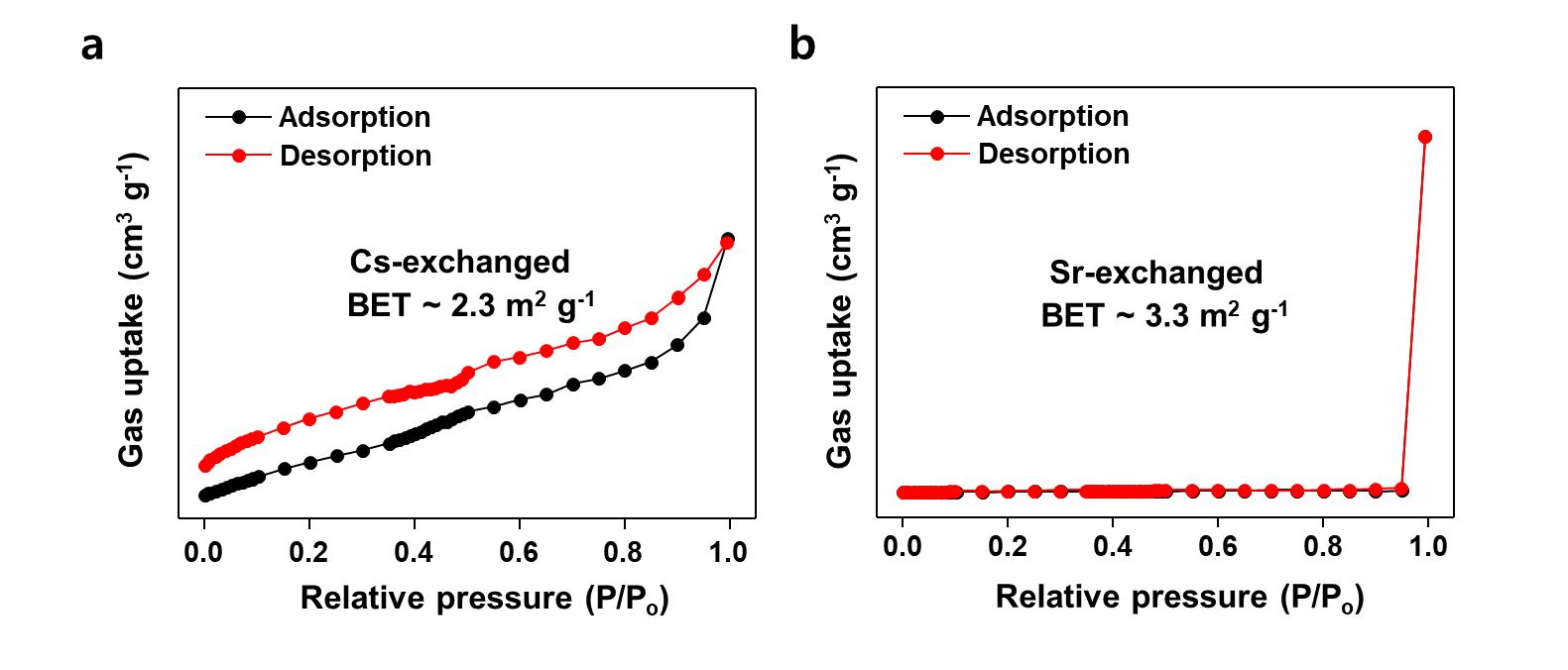
**

Supplementary Figure 28. **Pore-structure analysis of NMSC-1 after Cs^+^ and Sr^2+^ ion exchanges.** Brunauer–Emmett–Teller (BET) measures of NMSC-1 for **a** Cs-exchanged and **b** Sr-exchanged. Source data are provided as a Source Data file.

**
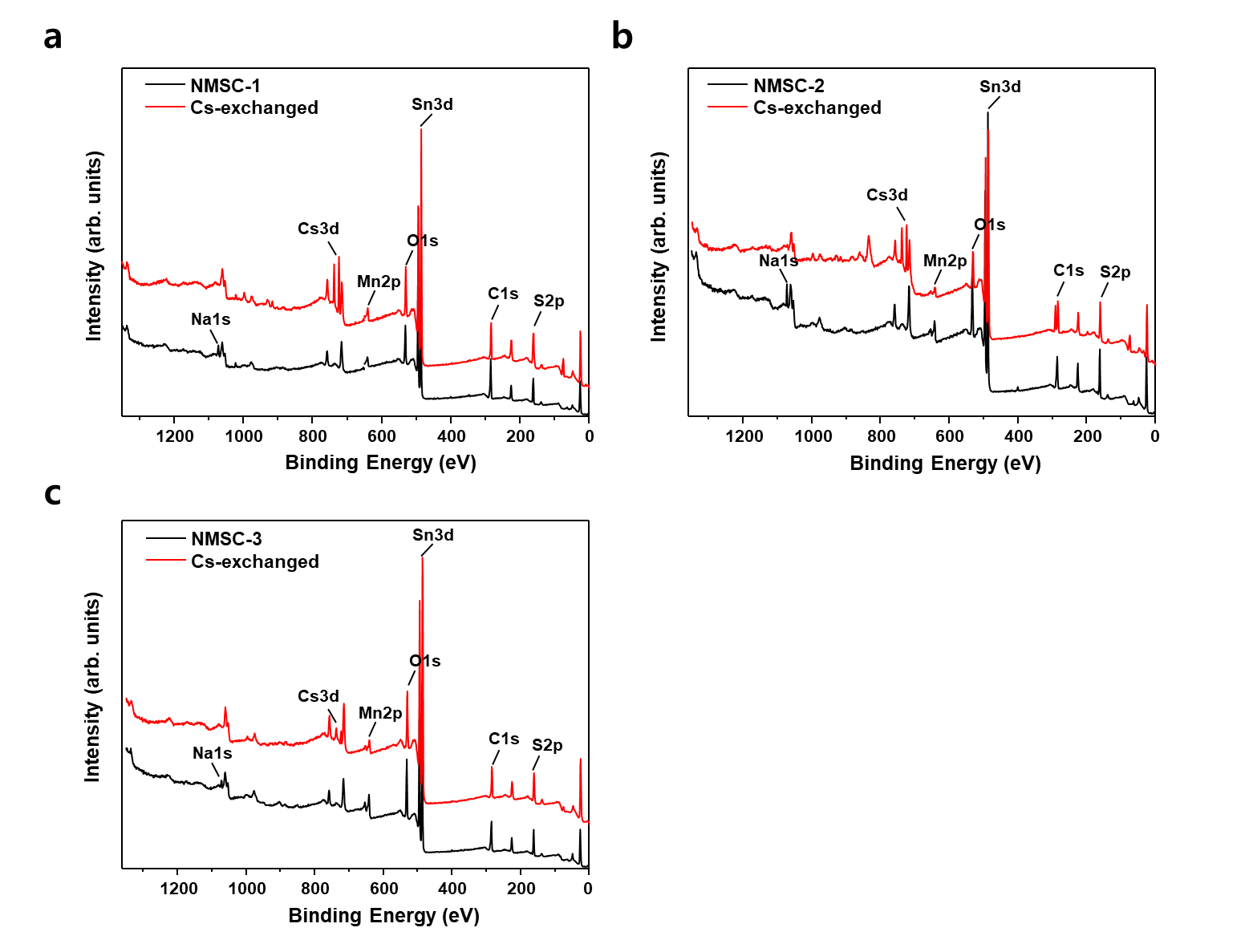
**

Supplementary Figure 29. XPS spectra of NMSC samples before and after Cs^+^ exchange. **a** NMSC-1, **b** NMSC-2, and **c** NMSC-3. Source data are provided as a Source Data file.

**
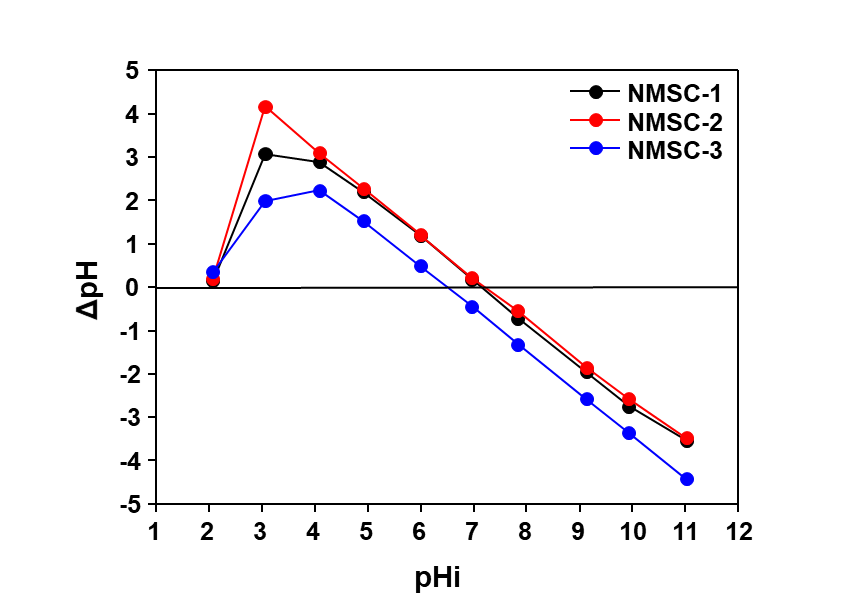
**

Supplementary Figure 30. ΔpH versus initial pH of NMSC suspensions at 298K. Source data are provided as a Source Data file.

Supplementary Tables

Supplementary Table 1**.** X-ray fluorescence (XRF) data of sodium manganese tin sulfide aerogels (NMSCs) for Na, Mn, Sn, and S elements, which indicate the inverse proportionality of manganese and sodium in different [Sn_2_S_6_]^4-^:Mn^2+^ ratios. Moreover, the amount of sulfur loss increases with residual acetate ion content in the Mn(OOCCH_3_)_2_ precursor.

| **Ratio**  **[Sn_2_S_6_]^4-^ : Mn^2+^** | **Labeled sample** | **Na** | **Mn** | **Sn** | **S** |
| --- | --- | --- | --- | --- | --- |
| 1.0 : 0.5 | NMSC-1 | 1.71 | 1.09 | 2.0 | 4.92 |
| 1.0 : 1.0 | NMSC-2 | 1.30 | 1.05 | 2.0 | 4.07 |
| 1.0 : 2.0 | NMSC-3 | 1.06 | 1.39 | 2.0 | 3.92 |

Supplementary Table 2. The BET Surface area values of NMSCs.

| **Samples** | **BET surface area (m^2^ g^-1^)** | | | **Average** | |
| --- | --- | --- | --- | --- | --- |
| NMSC-1 | 73 | 96 | 115 | | 95 |
| NMSC-2 | 101 | 114 | 156 | | 124 |
| NMSC-3 | 200 | 229 | 250 | | 226 |

Supplementary Table 3. Cs^+^ and Sr^2+^ removal capacities of NMSC samples and selected high-performance materials.

| **Materials** | **Adsorbate** | **C_i_** | **Q_e_** | **t (eq.)** | **Ref.** |
| --- | --- | --- | --- | --- | --- |
|  |  | **ppm** | **mg·g^-1^** | **min** |  |
| **KMS-1** | Cs^+^ | 1.1 | 226 | 60 | ^1^ |
| **CdSnSe-1** | Cs^+^ | 824 | 371.4 | 1440 | ^8^ |
|  | Sr^2+^ | 741 | 128.4 | 1440 |  |
| **PAN-KNiCF^d^** | Cs^+^ | 20~240 | 110.3 | 1440 | ^9^ |
| **GO-membrane on CaF_2_** | Cs^+^ | 87.3 | 148.0 | 30~1920 | ^10^ |
| **Graphene oxide** | Cs^+^ | 10 | 5.35 | 480 | ^11^ |
| **Na-GO fiber^e^** | Cs^+^ | 100 | 159.8 | 120 (10) | ^12^ |
| **Copper hexacyanoferrate (CuHCF)** | Cs^+^ | 100-200 | 155.60 | - | ^13^ |
|  | Sr^2+^ | 50-350 | 59.95 | - |  |
| **PB-GO hydrogel beads^a^** | Cs^+^ | 665 | 164.5 | 600(600) | ^14^ |
| **PSMGPB^b^** | Cs^+^ | 177300 | 213.9 | 1440(1440) | ^15^ |
| **UiO66-NH–SO3H-3^c^** | Cs^+^ | 60 | 118.76 | 90 | ^16^ |
|  | Sr^2+^ | 60 | 113.12 | 90 |  |
| **TAC-3** | Cs^+^ | 100 | 191.1 | 180 | ^4^ |
| **NMSC-1** | Cs^+^ | 100 | 78 (average) | 40 (180) | This study |
|  | Sr^2+^ | 100 | 41 (average) | (180) |  |
| **NMSC-2** | Cs^+^ | 100 | 40 (average) | - |  |
|  | Sr^2+^ | 100 | 34 (average) | - |  |
| **NMSC-3** | Cs^+^ | 100 | 34 (average) | - |  |
|  | Sr^2+^ | 100 | 23 (average) | - |  |
| Q_e_ = equilibrium adsorption capacity. C_i_ = initial Cs^+^ concentration. t (eq.) = sorption time (equilibrium time)  ^a^PVA-alginate encapsulated PB-GO hydrogel beads. ^b^pectin-stabilized magnetic graphene oxide Prussian blue nanocomposites ^c^UiO-66-NH2 with –SO_3_H functional ^d^PAN-based potassium nickel hexacyanoferrate (II) composite spheres. ^e^NaOH 2wt%, 500 um, 20 mg/ml, thermal treatment 3h | | | | | |

Supplementary Table 4. Atomic % of NMSC-1 after Cs^+^ and Sr^2+^ ion exchanges with absence/reduction of Na.

| **Element** | **Element & Atomic %** | | | | | |
| --- | --- | --- | --- | --- | --- | --- |
|  | **Na** | **Cs** | **Sr** | **Mn** | **Sn** | **S** |
| **Cs-exchanged** | 1.73 | 10.31 | - | 13.58 | 30.21 | 44.17 |
| **Sr-exchanged** | 0.61 | - | 3.20 | 12.44 | 36.77 | 46.99 |

Supplementary Table 5. Atomic % by XPS analysis of NMSC samples before and after Cs^+^ ion exchange.

| **Sample** | **Name & Atomic %** | | | | | | |
| --- | --- | --- | --- | --- | --- | --- | --- |
|  | **Na1s** | **Cs3d** | **Mn2p** | **O1s** | **Sn3d** | **C1s** | **S2p** |
| **NMSC-1** | 3.07 | - | 2.39 | 20.67 | 11.85 | 46.61 | 15.42 |
| **Cs^+^ exchanged** | - | 3.02 | 2.48 | 21.65 | 13.85 | 34.63 | 24.36 |
| **NMSC-2** | 3.09 | - | 3.65 | 23.42 | 13.72 | 34.24 | 19.60 |
| **Cs^+^ exchanged** | 0.52 | 3.19 | 2.47 | 17.96 | 14.05 | 44.54 | 17.28 |
| **NMSC-3** | 1.58 | - | 5.22 | 29.00 | 11.96 | 38.15 | 14.09 |
| **Cs^+^ exchanged** | 0.1 | 1.23 | 3.58 | 27.28 | 18.02 | 32.23 | 17.56 |

Supplementary References

1. Manos M. J., Kanatzidis M. G. Highly efficient and rapid Cs^+^ uptake by the layered metal sulfide K_2x_Mn_x_Sn_3−x_S_6_ (KMS-1). *J. Am. Chem. Soc.* **131**, 6599-6607 (2009).

2. Manos M. J., Ding N., Kanatzidis M. G. Layered metal sulfides: exceptionally selective agents for radioactive strontium removal. *Proc. Natl. Acad. Sci. U.S.A* **105**, 3696-3699 (2008).

3. Wang W., Li T., Ye K., Long H. B., Wang X., Ru H. Preparation of hierarchically mesoporous silica microspheres with ordered mesochannels and ultra-large intra-particulate pores. *Microporous Mesoporous Mater.* **234**, 267-276 (2016).

4. Kang Y. K.*, et al.* Thiostannate coordination transformation-induced self-crosslinking chalcogenide aerogel with local coordination control and effective Cs^+^ remediation functionality. *J. Mater. Chem. A* **8**, 3468-3480 (2020).

5. Gupta K., Yuan B., Chen C., Varnakavi N., Fu M.-L. K_2x_Mn_x_Sn_3−x_S_6_ (x= 0.5–0.95)(KMS-1) immobilized on the reduced graphene oxide as KMS-1/r-GO aerogel to effectively remove Cs^+^ and Sr^2+^ from aqueous solution. *Chem. Eng. J. (Amsterdam, Neth.)* **369**, 803-812 (2019).

6. Agasti N., Kaushik N. One pot synthesis of crystalline silver nanoparticles. *Am. J. nanomater.* **2**, 4-7 (2014).

7. Li J., Kessler H., Delmotte L. Study of novel mesostructured materials based on tin (IV) sulfide Part 1.—Synthesis and characterization. *J. Chem. Soc., Faraday trans.* **93**, 665-668 (1997).

8. Zhu J.-Y.*, et al.* Structural investigation of the efficient capture of Cs^+^ and Sr^2+^ by a microporous Cd–Sn–Se ion exchanger constructed from mono-lacunary supertetrahedral clusters. *Inorg. Chem. Front.*, (2022).

9. Du Z., Jia M., Wang X. Cesium removal from solution using PAN-based potassium nickel hexacyanoferrate (II) composite spheres. *J. Radioanal. Nucl. Chem.* **298**, 167-177 (2013).

10. Narayanam P. K., Jishnu A., Sankaran K. Graphene oxide supported filtration of cesium from aqueous systems. *Colloids Surf. A Physicochem. Eng. Asp.* **539**, 416-423 (2018).

11. Xing M., Zhuang S., Wang J. Efficient removal of Cs (I) from aqueous solution using graphene oxide. *Prog. Nucl. Energy* **119**, 103167 (2020).

12. Lee H., Lee K., Kim S. O., Lee J.-S., Oh Y. Effective and sustainable Cs^+^ remediation via exchangeable sodium-ion sites in graphene oxide fibers. *J. Mater. Chem. A* **7**, 17754-17760 (2019).

13. Le L. H. T.*, et al.* Prussian blue analogues of A_2_[Fe(CN)_6_] (A: Cu^2+^, Co^2+^, and Ni^2+^) and their composition-dependent sorption performances towards Cs^+^, Sr^2+^, and Co^2+^. *J. Nanomater.* **2021**, (2021).

14. Jang J., Lee D. S. Enhanced adsorption of cesium on PVA-alginate encapsulated Prussian blue-graphene oxide hydrogel beads in a fixed-bed column system. *Bioresour. Technol.* **218**, 294-300 (2016).

15. Kadam A. A., Jang J., Lee D. S. Facile synthesis of pectin-stabilized magnetic graphene oxide Prussian blue nanocomposites for selective cesium removal from aqueous solution. *Bioresour. Technol.* **216**, 391-398 (2016).

16. Wu J.*, et al.* Efficient removal of Sr^2+^ and Cs^+^ from aqueous solutions using a sulfonic acid-functionalized Zr-based metal–organic framework. *J. Radioanal. Nucl. Chem.* **328**, 769-783 (2021).
